# Developments of Thermosensitive Hydrogel for Maxillofacial Bone Regeneration

**DOI:** 10.1002/smsc.202500225

**Published:** 2025-07-22

**Authors:** Qinrou Zhang, Ying Wu, Jialiang Dai, Zihan Jia, Minjia Zhu, Xinyi Li, Le Xiao, Jingyi Li, Zixiang Dai, Yuxing Bai, Jibin Song, Ke Zhang

**Affiliations:** ^1^ Department of Orthodontics Beijing Stomatological Hospital, School of Stomatology Capital Medical University Beijing 100070 China; ^2^ State Key Laboratory of Chemical Resource Engineering College of Chemistry Beijing University of Chemical Technology Beijing 100029 China; ^3^ Department of Stomatology Beijing Friendship Hospital Capital Medical University Beijing 100050 China

**Keywords:** bone regeneration, bone tissue engineering, drug delivery system, maxillofacial bone, thermosensitive hydrogels

## Abstract

Maxillofacial bone defects usually lead to facial deformities and multifunctional impairments, which tremendously damage patients’ mental health and social functioning. Recently, the thermosensitive hydrogel combined with seed cells and growth factors has offered a novel approach for maxillofacial bone regeneration. Remarkably responsive to external temperature changes, thermosensitive hydrogel can transform between sol and gel states. Furthermore, its unique properties, such as fluidity, minimally invasive injectability, localized applicability, and controllable drug release, have been increasingly recognized, endowing it with significant promise in bone regeneration. Cutting‐edge research on the effects of thermosensitive hydrogels has been reviewed. However, the mechanisms involved in promoting bone regeneration in the maxillofacial region have not yet been established. This article represents the first review of the specific mechanism of thermosensitive hydrogel in angiogenesis and neurogenesis, specifically focusing on its role in maxillofacial osteogenesis. Finally, the article examines the scaffold and drug delivery capabilities of thermosensitive hydrogel in maxillofacial osteogenesis. This review is expected to provide some insights into the advanced developments of thermosensitive hydrogel for maxillofacial bone regeneration.

## Graphical abstract

Thermosensitive hydrogel with seed cells and growth factors presents a new method for bone regeneration, changing states with temperature and offering fluidity, injectability, and controlled drug release. This article reviews thermosensitive hydrogel's role in angiogenesis, neurogenesis, and osteogenesis in maxillofacial bone regeneration and discusses its scaffold and drug delivery capabilities, aiming to ‌illuminate‌ its advancements in bone regeneration.

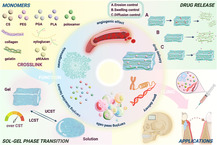

## 1 Introduction

Hydrogel is an aqueous, viscous, semi‐solid material prepared through physical or chemical crosslinking, consisting of a gel matrix and loaded substances. Due to its excellent characteristics, such as good biocompatibility, biodegradability, and nontoxicity, hydrogel is widely utilized in various biomedical fields, including drug delivery, wound dressing, tissue engineering, biosensing, bioprinting, and electrospinning.^[^ ^1^ ^]^ In recent years, stimuli‐responsive hydrogels have garnered increasing attention.^[^ ^2^ ^]^ They are capable of inducing variations in the local characteristics of polymers, triggered by external physical stimuli such as light, temperature, electric and magnetic fields, as well as by intrinsic chemical or biological stimuli inherent in the local microenvironment, including pH shifts, glucose concentrations, reactive oxygen species, and enzyme activities.^[^ ^3^ ^]^ Among these, thermosensitive hydrogels are highly promising candidates for biomedical applications. When the polymer solution of this type of hydrogel is exposed to temperatures above or below a specific critical solution temperature (CST), phase separation occurs. Its physical state can change with temperature variations, leading to a transition between sol and gel states.^[^ ^4^ ^]^

By adjusting the composition ratio of their internal matrix, thermosensitive hydrogels can achieve specific response temperatures and response times.^[^ ^5^ ^]^ Remarkably, some thermosensitive polymers can undergo a sol–gel transition at 37 °C, enabling the formation of hydrogels in situ and allowing the encapsulation of drugs and therapeutics under body temperature conditions. This feature makes them highly suitable for biomedical applications in the human body.^[^ ^6^ ^]^ Once the in situ gel is formed, it serves as a repository for drug delivery, offering benefits like microinvasiveness, localized application, and controllable drug release. This approach can reduce systemic toxicity, decrease administration frequency, and ease patient compliance requirements.^[^ ^7^ ^]^

The maxillofacial region comprises several nonload‐bearing elements abundantly supplied with blood, and its bone defects often feature small, irregular forms.^[^ ^8^ ^]^ The compressive strength and Young's modulus of cancellous bone range from 2 to 6 MPa and 0.1 to 0.3 GPa, respectively.^[^ ^9^ ^]^ This index can be achieved by adjusting the proportion of each component within the thermosensitive hydrogel. The gel in a solution state possesses fluidity, enabling it to effortlessly fill every nook and cranny of these defects, particularly the irregular ones. Furthermore, maxillofacial bone defects pose a significant risk of microbial infection within the oral cavity, and the soft tissue envelope and stem cells surrounding the defect are inadequate.^[^ ^10^ ^]^ Some gels can even be locally injected through syringes, which boasts minimal invasiveness and simplicity in operation. Upon curing, the gel facilitates the growth of stem cells into the available space and gradually releases active factors and drugs. Consequently, many scholars are dedicating their efforts to investigating hydrogels for maxillofacial bone tissue repair and regeneration.^[^ ^11^ ^]^

**Figure 1:**
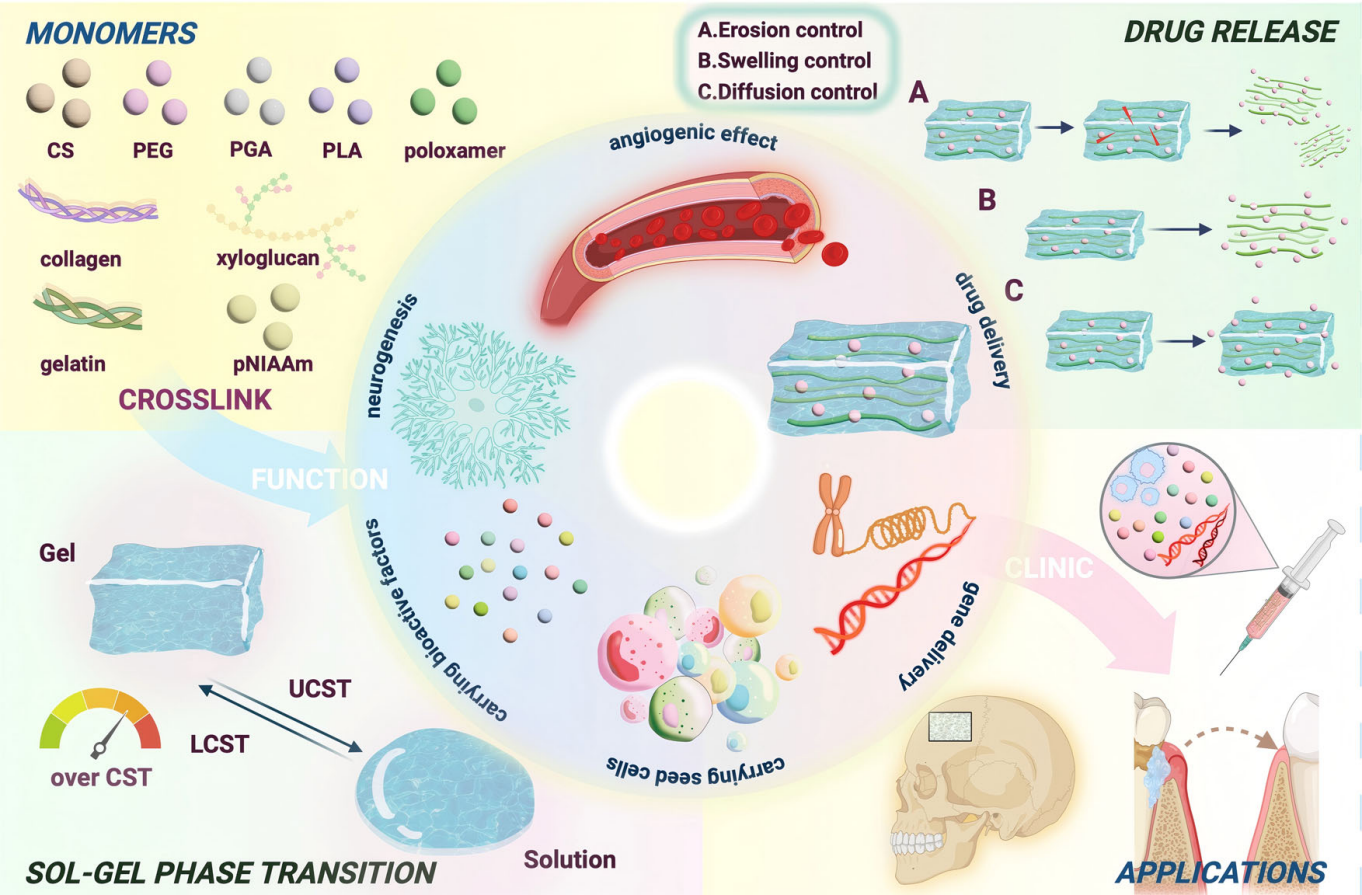
Schematic illustration of thermosensitive hydrogel in maxillofacial bone regeneration. Thermosensitive hydrogels can be synthesized from a range of substances, such as chitosan (CS), poly(ethylene glycol)(PEG), poly(glycolic acid)(PGA), poly(lactic acid)(PLA), poloxamer, collagen, xyloglucan, gelatin, and poly(N‐isopropylacrylamide)(pNIPAAm), using physical or chemical crosslinking methods, resulting in a phase transition at the critical temperature. Moreover, these hydrogels have significant potential in promoting angiogenesis, neurogenesis, and osteogenesis through their inherent material properties as drug delivery vehicles or by providing suitable mechanical support. Furthermore, by constructing a drug delivery system, thermosensitive hydrogels can effectively and efficiently deliver drugs as needed through various mechanisms, including erosion, swelling, and diffusion control. Thus far, thermosensitive hydrogels have garnered considerable attention in the applications of maxillofacial bone regeneration (created with BioRender.com).

While predecessors have examined certain functions of thermosensitive hydrogels, a comprehensive understanding of their mechanism and clinical application in craniofacial osteogenesis remains elusive. This review marks the first exploration of thermosensitive hydrogels in maxillofacial bone regeneration. It delves into the intricate mechanisms of these hydrogels, including their neurogenic and angiogenic effects, as well as their potent capacity to stimulate bone formation. Additionally, the review provides comprehensive insights into drug delivery methods that utilize these hydrogels (**Figure** **1**).

## 2 Construction of Thermosensitive Hydrogel

Hydrogels consist of 3D crosslinked networks formed by the interconnection of hydrophilic polymer chains. Minor temperature variations near the CST trigger either the collapse or relaxation of the polymer chains within the hydrogel due to adjustments in the interactions between the hydrogel's polymer chains and the surrounding water molecules.^[^ ^12^ ^]^ It can be categorized into the upper critical solution temperature (UCST) and the lower critical solution temperature (LCST).^[^ ^1^ ^]^ In the case of UCST, a thermosensitive polymer undergoes a phase transition in environments below this temperature. Polymers with UCST exhibit solubility above the threshold temperature and gelation below it. Conversely, LCST is the opposite of UCST.

Thermosensitive polymers exhibiting UCST include carrageenan, starch, agarose, and gellan gum.^[^ ^4^ ^]^ However, there are still few studies on thermosensitive polymers with UCST in bone regeneration. Their limited use in biomedicine arises from reaching the solution state at temperatures exceeding physiological levels. Recently, some UCST polymers with a temperature range close to that of the human body have been created in another field, for instance, poly(acrylamide*‐co*‐acrylonitrile), which has a UCST between 30 and 50 °C.^[^ ^13^, ^14^ ^]^ In bioelectronics, the UCST of materials can be decreased from 58 to 19 °C through precise manipulation of the quaternized chitosan and acrylic acid monomer ratio.^[^ ^15^ ^]^ In the field of wound healing, UCST can be employed to mimic the physiological response of human pores upon heating.^[^ ^16^ ^]^ Due to UCST's contrasting transition, further exploration is required for potential future applications.

In contrast, thermosensitive polymers with LCST have garnered significant attention. At the molecular level, the hydrophobic groups of these polymers interact at elevated temperatures, leading to the collapse of exposed water molecules within the polymer chain. Simultaneously, the hydrogel's thermal response induces changes in swelling. This characteristic is highly significant in pharmacology, particularly drug delivery applications.^[^ ^12^ ^]^

Thermosensitive hydrogels can be produced through either physical or chemical crosslinking methods. Physical crosslinking is a prevalent approach in hydrogel fabrication. This network structure is stabilized by various interactions, including hydrogen bonding, π–π stacking, dipole–dipole forces, van der Waals forces, and hydrophobic interactions. Notably, physical crosslinking does not require the use of crosslinking agents, thereby avoiding potential complications such as unreacted monomers, initiators, or exothermic reactions that could cause unintended damage to the application site. Moreover, physically crosslinked hydrogels offer a hospitable environment for cells, biomacromolecules, and bioactive factors.^[^ ^17^ ^]^ Simultaneously, they exhibit diverse attributes, including size, shape, structure, and chemical/physical functionalities, enabling precise control over the timing and location of drug release while ensuring drug stability.^[^ ^18^ ^]^

On the other hand, hydrogels synthesized via chemical crosslinking rely on the formation of covalent bonds between polymer chains, often resulting in irreversible bonding. Consequently, these hydrogels exhibit superior mechanical strength and stability. Standard chemical crosslinking techniques include free‐radical polymerization, Michael addition reactions, photopolymerization, enzymatic reactions, and disulfide bond formation.^[^ ^19^ ^]^ Regarding chemical crosslinking, the primary emphasis lies in synthesizing precursors for physically crosslinked hydrogels.^[^ ^20^ ^]^

The thermosensitive hydrogel can serve as the polymer backbone for the latest DNA cryogel. By harnessing its phase transition properties, the hydrogel's backbone network collapses when the temperature exceeds the LCST, revealing the hydrophilic DNA strand and allowing it to bind to specific cells. Conversely, the backbone network expands when the temperature falls below the LCST, enclosing the DNA strand and releasing the cells. This mechanism enables the construction of a temperature‐controlled cell capture and release system.^[^ ^21^ ^]^ The thermosensitive hydrogel can also be integrated with 3D printing technology.^[^ ^22^, ^23^ ^]^ Certain thermosensitive materials, like gelatin microrods, can serve as pore‐forming agents. These agents dissolve at body temperature, creating a microchannel structure within the gel that mimics the pore structure found in human tissues.^[^ ^24^ ^]^ The phase transition properties of the thermosensitive hydrogel make it ideal for application on the surface of metal implants. The hydrogel's ample hydration layer effectively prevents bacterial adhesion at room temperature. However, the hydrogel structure collapses when exposed to body temperature, revealing a porous texture that traps water, reduces bacterial binding sites, and promotes cell adhesion and proliferation.^[^ ^25^ ^]^ Certain studies have observed scholars utilizing the unique characteristics of thermosensitive hydrogels in reverse. By releasing drugs at body temperature and increasing drug release when body temperature abnormally drops, these hydrogels can serve the dual purpose of detection and protection.^[^ ^26^ ^]^

Due to the characteristics mentioned above, the thermosensitive hydrogel demonstrates distinct characteristics in comparison to other responsive gels (**Table** **1**) and boasts a broad spectrum of applications across various fields, diseases, and bodily parts, including deep tissue imaging,^[^ ^27^ ^]^ wound dressing,^[^ ^28^ ^]^ intravascular,^[^ ^29^ ^]^ intrauterine,^[^ ^30^ ^]^ and ocular regions.^[^ ^31^ ^]^ The maxillofacial region comprises numerous nonload‐bearing components, boasts a rich blood supply, and features minor, irregular bone defects; thus, thermosensitive hydrogels exhibit significant application advantages. The application of thermosensitive hydrogels in maxillofacial bone regeneration will be further elaborated.

**Table 1:** Different externally stimuli‐responsive hydrogels for maxillofacial bone regeneration.^[^ ^119^, ^120^, ^121^, ^122^, ^123^, ^124^, ^125^, ^126^, ^127^, ^128^, ^129^, ^130^, ^131^, ^132^, ^133^, ^134^ ^]^

Hydrogel Type	Molecular Mechanism	Mechanical Properties	Drug Release Kinetics	In Vivo Performance
Thermosensitive	Hydrophobic and hydrophilic groups (e.g., poly(N‐isopropyl acrylamide) (pNIPAApm), polysaccharides, and polypeptides)	Limited strength Reversible gelation (near CST) Adjustable CST Excellent injectability	Temperature‐triggered The temperature can be secondarily controlled by other particles in the gel	Defect‐conforming gelation Tunable degradation
Photo‐responsive	Photo‐caging groups (e.g., coumarin and azobenzene)	Adjustable modules Rapid curing Suit for 3D printing	Photo‐triggered release	Controlled release of deep tissue Tissue‐related light attenuation and phototoxicity
pH‐responsive	Weak acid or basic groups on polymer chains (e.g., poly(acrylic acid) (pAA) and polyacrylamide (pAAm))	Unstable mechanical strength	Pathology‐targeted release pH‐dependent release rate	Early detection Suitable for pH‐sensitive pathological conditions
Magneto‐responsive	Magnetocaloric effect/magnetic drive	Modulus can be tuned by introducing magnetic particles, such as ferroferric oxide	Targeted drug delivery Magnetocaloric effect for therapeutic applications	Magnetic field‐guided osteogenesis
Electroresponsive hydrogel	The movement of ions in response to an external electric field and the rearrangement of these ions (e.g., polypyrrole (PPy), polyaniline (PANI))	Adjustable modules Requires conductivity	Electrically driven motility	Potential damage when applying electric current Inadequate biocompatibility
Ultrasound‐responsive (e.g., polymerizing N,N‐dimethylacrylamide (DMA), and methacrylic acid (MAA))	Acoustic cavitation/thermal effect	Adjustable modules Ultrasound can affect the gel molecular chain	Complete ultrasound Stimulation control release Regulating ultrasonic parameters to regulate release	Bioimaging Suitable for deep jaw defects Lack of precise control Potential risk of organelles rupture
Mechano‐responsive	Reversible or irreversible conformational change, dynamic cross‐links or the non‐covalent interactions of the polymeric networks or the molecular chains	The intrinsic mechanical or chemical properties of hydrogels are changed by mechanical stimulation.	Stress‐triggered drug release	Detection and early warning Adapt to the dynamic mechanical environment of bone

## 3 Thermosensitive Hydrogel Promotes an Angiogenic Effect

Vascularization plays a pivotal role in the process of bone repair.^[^ ^32^ ^]^ During bone regeneration, the formation of blood vessels is crucial for supplying nutrients and oxygen, facilitating calcium salt deposition, regulating cell growth and differentiation, as well as removing metabolic waste.^[^ ^33^ ^]^ During epidermal healing, mechanical stretching stimulates the proliferation of keratinocytes via some distinct pathways, such as mitogen‐activated protein kinase 1/2 pathway, phosphoinositide 3‐kinase pathway, epidermal growth factor receptor, and calcium channels.^[^ ^34^ ^]^

**Figure 2:**
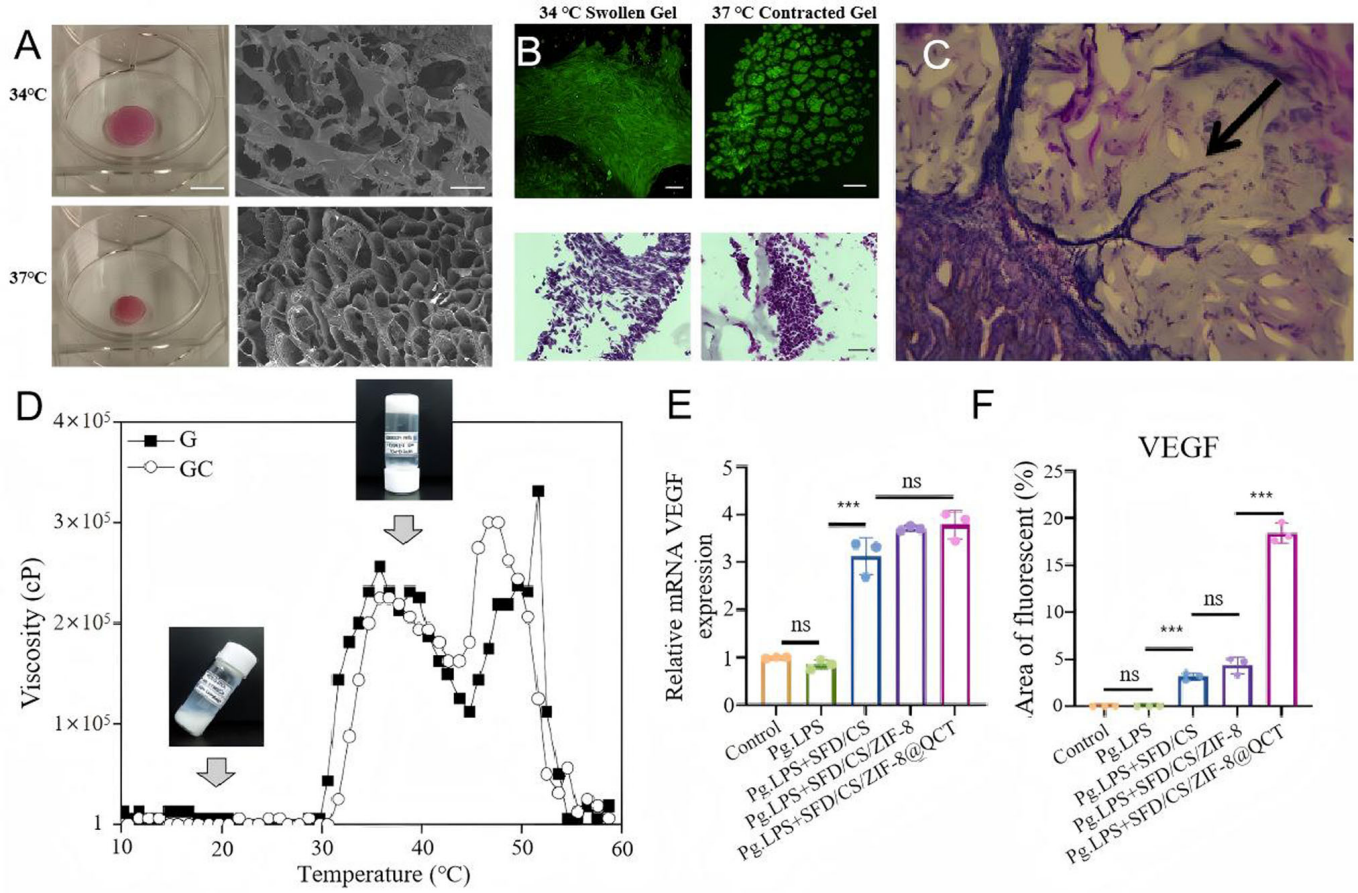
A) GRGDS‐pNIPAAm in swollen and contracted states at 34 and 37 °C, respectively (bar, 1 cm). Scanning electron microscopy (SEM) images of GRGDS‐pNIPAAm gels in swollen (top) and contracted (bottom state showing a reduction in pore size with gel contraction (bar, 100 μm). Reproduced with permission,^[^ ^35^ ^]^ Copyright 2014, Wiley. B) Fluorescent micrographs and light micrographs showing Hematoxylin‐Eosin staining of E10 DMC in swollen GRGDS‐pNIPAAm hydrogel at 34 °C and in contracted GRGDS‐pNIPAAm hydrogel at 37 °C (bar, 50 μm). Reproduced with permission,^[^ ^35^ ^]^ Copyright 2014, Wiley. C) Hematoxylin‐Eosin‐stained histological sections of DM cells in a contracted gel, and the arrow indicates a new capillary sprout (bar, 100 μm). Reproduced with permission,^[^ ^35^ ^]^ Copyright 2014, Wiley. D) Viscosity versus temperature curves for injectable 20 wt% MP solutions without (G) or with (GC) hTMSCs. Reproduced with permission,^[^ ^36^ ^]^ Copyright 2014, Elsevier. E) Relative mRNA expression of angiogenesis‐related gene (VEGF) of PDLSCs on day 7 measured by RT‐qPCR. Reproduced with permission,^[^ ^37^ ^]^ Copyright 2024, KeAi. F) The immunofluorescence staining and its quantitative analysis of angiogenesis‐related protein (VEGF) of macrophages on day 7. Reproduced with permission,^[^ ^37^ ^]^ Copyright 2024, KeAi.

The contraction of the thermosensitive hydrogel can generate the mechanical tensile force on cells, thereby fostering angiogenesis. A porous hydrogel scaffold was created using GRGDS‐modified pNIPAAm (**Figure** **2**A). This hydrogel remains expanded in the cold and rapidly contracts volumetrically when exposed to body temperature.^[^ ^35^ ^]^ When undifferentiated embryonic dental mesenchymal cells (DMC) were seeded within this hydrogel and polymer shrinkage was thermally induced by warming, the cells became physically compressed and exhibited a more compact and rounded morphology similar to that observed during mesenchymal condensation in tooth organ development within the embryo (Figure 2B). Histological analysis of these hydrogels implanted for two weeks under the kidney capsule of a mouse revealed that only the contracted gel‐containing cells encapsulated within the shrink‐wrapped GRGDS‐pNIPAAm polymer induced neovascularization (Figure 2C).

Methoxy polyethylene glycol‐polycaprolactone block copolymer (MPEG‐PCL(MP)) has been experimentally tested as an injectable in situ‐forming hydrogel. The MP solution loaded with human turbinate mesenchymal stromal cells (hTMSCs) became a hydrogel at 37 °C in no more than 10 s (Figure 2D).^[^ ^36^ ^]^ Some host cells and a few new blood vessels were observed within all the hydrogels.

Another thermosensitive hydrogel was constructed by dopamine‐modified silk fibroin (SFD) and the classic thermosensitive hydrogel (CS/β‐glycerophosphate (β‐GP)). It demonstrated free‐flowing characteristics at 4 °C and quickly (≈2 min) transformed into hydrogels at 37 °C.^[^ ^37^ ^]^ This system can load zeolitic imidazolate framework‐8 (ZIF8) and quercetin (QCT). Cocultured PDLSCs with composite hydrogels for 7 days, the addition of composite hydrogel significantly boosted the expression of the angiogenic gene (VEGF) (Figure 2E). Moreover, they exhibit favorable cytocompatibility for human umbilical vein endothelial cells (HUEVC). The SFD/CS/ZIF‐8 and SFD/CS/ZIF8@QCT induced significantly more tube formation (Figure 2F) and markedly enhanced the angiogenic gene expression (bFGF, VEGF, and vWF) of HUVEC, with the latter exhibiting a more pronounced effect than the former.

Bone tissue scaffolds with exceptional vascular regeneration capabilities are crucial for ensuring adequate oxygen and nutrient exchange, which is vital for bone regeneration.^[^ ^38^ ^]^ The primary hurdle in vascularizing bone tissue engineering lies in replicating the intricate structure of the natural vascular network found in living organisms. Typically, two methods are employed: constructing microchannels within scaffolds to facilitate the exchange of nutrients and oxygen and promoting angiogenesis by incorporating vascular growth factors or cells.^[^ ^39^, ^40^ ^]^ Ultimately, both approaches rely on endogenous vascularization. Currently, there is no consensus on the ideal scaffold diameter for osteogenesis and vascularization, with reported ranges varying from 100–350, 200–400, and 400–600 μm.^[^ ^41^ ^]^ The thermosensitive hydrogel can physically alter cell morphology via contraction and expansion, while the in situ hydrogel can enhance the angiogenic effect by collaborating with various components. It establishes a beginning part of bone regeneration and provides a robust foundation for subsequent neurogenesis and bone regeneration processes.

## 4 Thermosensitive Hydrogel Promotes Neurogenesis

Bones are richly innervated by peripheral nerves, and nerve regeneration plays a crucial role in bone regeneration.^[^ ^42^ ^]^ Neurotransmitters, neuropeptides, and nerve cell redifferentiation are the primary features of neuro‐skeletal regulation and have complex molecular mechanisms.^[^ ^43^ ^]^ Effective neural regulation can control bone homeostasis and promote regeneration.^[^ ^44^ ^]^ This aspect has also been gaining increasing attention.^[^ ^45^, ^46^, ^47^, ^48^ ^]^ An innovative concept, the nerve–bone axis, has been introduced.^[^ ^49^ ^]^ According to this concept, nerves and bones can mutually regulate each other via paracrine and autocrine signaling.^[^ ^50^, ^51^ ^]^ For instance, the sensory nerve can regulate skeletal effects by gene‐related peptide, substance P, and peptidyl‐prolyl *cis*‐*trans* isomerase D.^[^ ^52^, ^53^, ^54^ ^]^ Meanwhile, some axon guidance molecules and neurotrophins like semaphorin3A, semaphorin4D, nerve growth factor (NGF), and brain‐derived neurotrophic factor are the key players in bone remodeling and regeneration.^[^ ^49^ ^]^

The composite thermosensitive hydrogels (CRP) fabricated using CS, RADA_16_ nanofibers, and nerve‐promoting peptide (PPFLMLLKGSTR) can form an elastic hydrogel network at 37 °C.^[^ ^55^ ^]^ It not only has the capability to enhance the proliferation and migration of bone marrow mesenchymal stem cells (BMSCs), but it also demonstrates the ability to stimulate the proliferation and differentiation of neural stem cells into neurons.^[^ ^55^ ^]^ Simultaneously, it may activate the PI3K/AKT/mTOR pathway to repair spinal cord injury.^[^ ^55^ ^]^ Another soft thermosensitive polymer electroactive hydrogel loaded with NGF can release NGF about 87.02% ± 3.05% of the lysozyme within 24 days and degraded 4 weeks in vivo.^[^ ^56^ ^]^ In different thermosensitive hydrogels, such as PF127, it exhibited a two‐stage release pattern, with fast release NGF in the first 10 h, followed by sustained release in the last 8 h.^[^ ^57^ ^]^

In some nonthermosensitive hydrogels, hydrogels composed of hyaluronic acid and adipic acid dihydrazide, loaded with gingering‐derived small extracellular vesicles (sEV) and Mg^2+^, can recruit bone marrow‐derived mesenchymal stem cells to the wound sites and stimulate them toward neurogenic differentiation. This leads to robust angiogenesis which allows a regenerative neurogenesis–angiogenesis cycle.^[^ ^58^ ^]^ Adding magnesium ion‐modified black phosphorus nanosheets into gelatin methacryloyl (GelMA) hydrogel presented multiple advantages for the upregulation of nerve‐related protein expression in neural stem cells.^[^ ^59^ ^]^ GelMA containing copper ion‐modified germanium–phosphorus was found to enhance angiogenesis and neurogenesis, eventually contributing to rat calvarial bone regeneration.^[^ ^60^ ^]^

**Figure 3:**
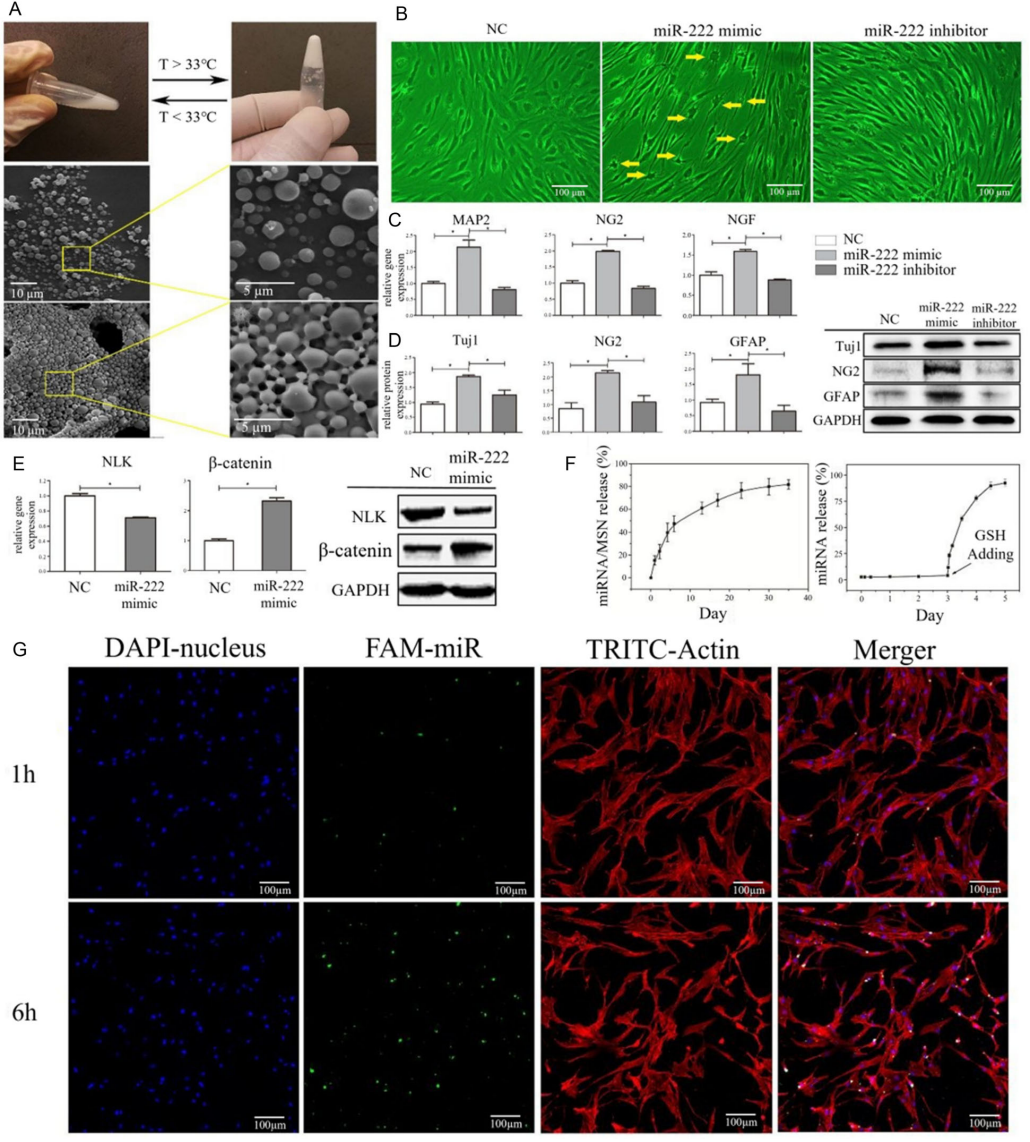
A) Photographs of the thermal responsive microspheres–hydrogel transition and the SEM images of corresponding microspheres in solution state and hydrogel state. Reproduced with permission,^[^ ^61^ ^]^ Copyright 2019, Royal Society of Chemistry. B) Branched and neural‐like morphology of hBMSC in the miR222 mimic group (yellow arrow). Reproduced with permission,^[^ ^61^ ^]^ Copyright 2019, Royal Society of Chemistry. C) Expression of neuronal markers MAP2, NG2, and NGF in hBMSC of the miR222 mimic was elevated, compared with NC and inhibitor group as determined by qRT‐PCR. Reproduced with permission,^[^ ^61^ ^]^ Copyright 2019, Royal Society of Chemistry. D) Western blot analysis of the neuronal proteins Tuj1, NG2, and GFAP was increased in the miR222 mimic group, compared with the NC and inhibitor group, and the fold change of each protein relative to that in the NC group was quantified. Reproduced with permission,^[^ ^61^ ^]^ Copyright 2019, Royal Society of Chemistry. E) NLK and β‐catenin expression after transfection of miR222 mimic and NC as determined by qRT‐PCR and western blotting. Reproduced with permission,^[^ ^61^ ^]^ Copyright 2019, Royal Society of Chemistry. F) Release profile of miR222/MSN complexes for 35 days in vitro, and glutathione was required to trigger burst release of miR222. Reproduced with permission,^[^ ^61^ ^]^ Copyright 2019, Royal Society of Chemistry. G) Cellular uptake of carboxyfluorescein‐conjugated miR222 after 1 and 6 h, as determined by confocal analysis. Green: miR222/MSN; red: F‐actin; and blue: cell nucleus. Reproduced with permission,^[^ ^61^ ^]^ Copyright 2019, Royal Society of Chemistry.

An injectable thermosensitive hydrogel poly(ethylene glycol)‐b‐poly(lactic‐*co*‐glycolic acid)‐b‐poly(N‐isopropylacrylamide) (PEG‐PLGA‐PNIPAM) was loaded with mesoporous silica nanoparticle (MSN), microRNA‐222, and aspirin (**Figure** **3**).^[^ ^61^ ^]^ It could form a hydrogel at 37 °C for 5 min. miR222 could induce human bone mesenchymal stem cells from excised mandibular cancellous bone differentiation into neural‐like cells through Wnt/β‐catenin/Nemo‐like kinase signaling. miR222 was released from hydrogels and could be taken by cells. In a rat mandibular bone defect, injection of the codelivered MSN hydrogel resulted in neurogenesis and enhanced bone formation.

With the introduction of the nerve–bone axis concept, numerous studies have demonstrated a tight correlation between neuromodulation and bone homeostasis.^[^ ^62^ ^]^ At the same time, neurogenesis maintains a strong correlation with angiogenesis, modulating the multiplication and vascularization of endothelial cells, thereby facilitating osteogenesis.^[^ ^63^, ^64^ ^]^ The trifecta of research focusing on angiogenesis, neurogenesis, and osteogenesis has attracted increasing attention.^[^ ^43^, ^65^, ^66^ ^]^ However, there is a little research on thermosensitive hydrogels in neurogenesis in maxillofacial bone regeneration, which represents an emerging field ripe for exploration.

## 5 The Application of Thermosensitive Hydrogel in Bone Tissue Engineering

Bone tissue engineering has emerged as a beacon of hope for the repair of maxillofacial defects. This field revolves around three pivotal components: seed cells, scaffold materials, and bioactive factors.^[^ ^67^, ^68^, ^69^ ^]^

### 5.1 Carrying Seed Cells

Standard seed cells used in maxillofacial bone repair include BMSCs, periodontal ligament stem cells (PDLSCs),^[^ ^70^ ^]^ dental pulp stem cells (DPSCs),^[^ ^71^ ^]^ adipose‐derived stem cells (ASCs),^[^ ^72^ ^]^ and turbinate mesenchymal stromal cells (TMSCs).^[^ ^36^ ^]^

**Figure 4:**
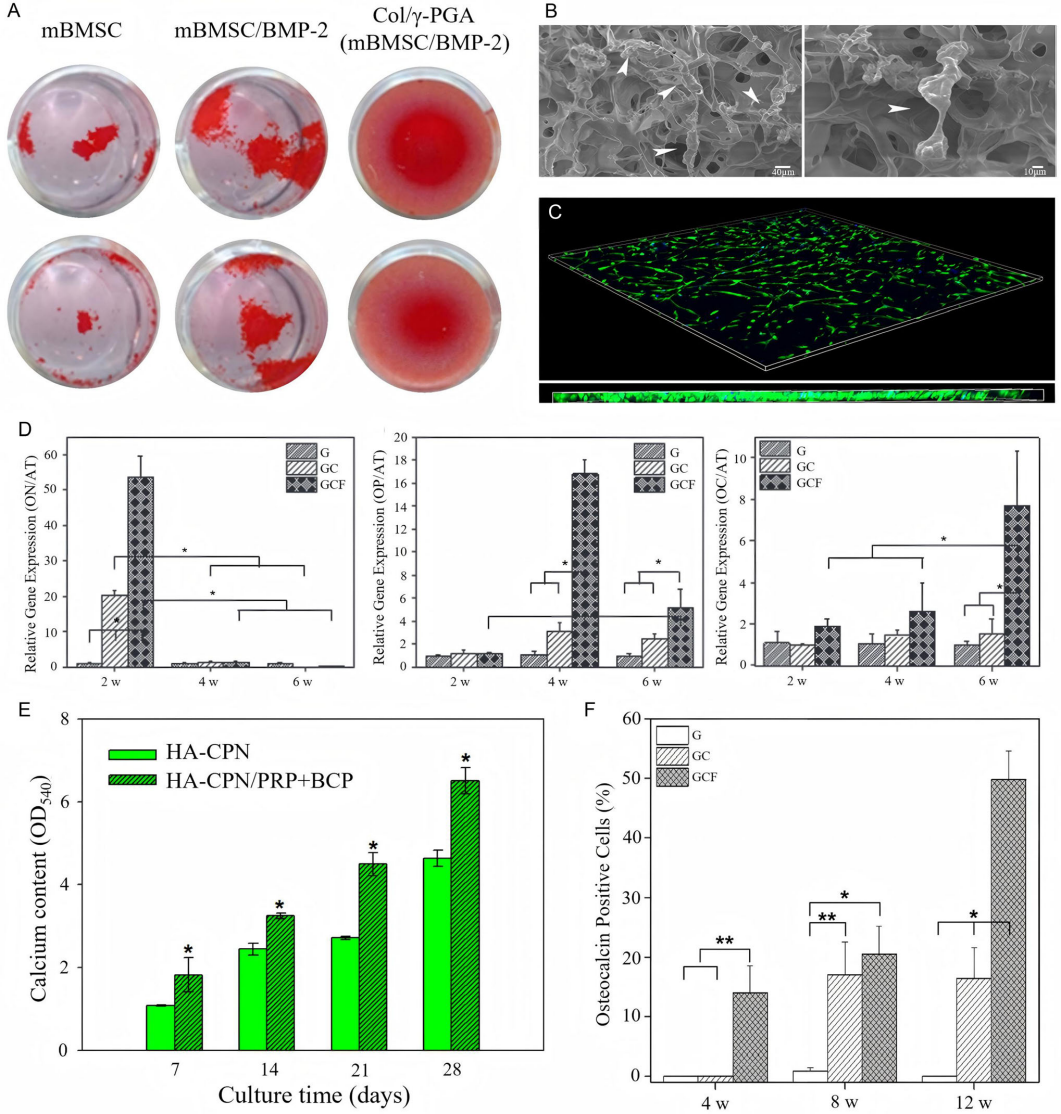
A) ARS staining at 7 days after culturing in differentiation medium on 24‐well plates. Reproduced with permission,^[^ ^73^ ^]^ Copyright 2022, Springer. B) SEM images demonstrating tight hPDLSCs adherence to and proliferation on hydrogel surfaces. White arrows indicate hPDLSCs. Reproduced with permission,^[^ ^75^ ^]^ Copyright 2019, Mary Ann Liebert Inc. C) hPDLSCs were grown for 7 days in the hydrogel matrix before Live/Dead staining and confocal microscopy. Green cells are alive, whereas red cells are dead. Blue corresponds to the cell nucleus. Reproduced with permission,^[^ ^75^ ^]^ Copyright 2019, Mary Ann Liebert Inc. D) Osteonectin (ON), osteopontin (OP), and osteocalcin (OC) gene expression in hydrogels removed from rats receiving G (PC only), GC (PC + hPDSCs), or GCF (PC + hPDSCs + osteogenic factors) after two, four, and six weeks. Statistical analysis was performed using one‐way ANOVA with Bonferroni's multiple comparison correction (*P < 0.001). Reproduced with permission,^[^ ^76^ ^]^ Copyright 2016, Wiley. E) The calcium content of rASC cultured in HA‐CPN and HA‐CPN/PRP/BCP thermogelling hydrogel scaffolds. *P < 0.05 compared with HA‐CPN. Reproduced with permission,^[^ ^72^ ^]^ Copyright 2018, MDPI. F) OC‐positive cells were counted on 3 images from each slide, and counts were averaged within each group. Statistical analysis was performed using one‐way ANOVA with Bonferroni's multiple comparison correction (*P < 0.001, **P < 0.01). Reproduced with permission,^[^ ^36^ ^]^ Copyright 2014, Elsevier.

The mouse BMSCs (mBMSCs) were loaded in collagen/poly‐c‐glutamic acid (Col/c‐PGA) hydrogel with bone morphogenetic protein (BMP)‐2 and showed good survival and proliferation ability (**Figure** **4**A).^[^ ^73^ ^]^ The hydrogel at 37 °C showed an open‐porous 3D interconnected structural cross‐section and effective orthotopic bone formation in a mouse model with a critical‐sized bone defect in only 3–4 weeks. The human BMSCs in chitosan‐poly(ethylene oxide) (PEO) hydrogel with recombinant human bone marrow protein‐2 (rhBMP‐2) also showed a high amount of bone regeneration in rat calvaria defects.^[^ ^74^ ^]^

The PDLSCs exhibit superior proliferation and osteogenic differentiation capabilities compared to BMSCs.^[^ ^70^ ^]^ The PDLSCs overexpressing PDGF‐BB were loaded with close adhesion in a PLGA‐PEG‐PLGA triblock copolymer (Figure 4B,C), which enhanced calcium deposition, osteogenic differentiation, and bone growth in the context of alveolar bone defects.^[^ ^75^ ^]^

The human DPSCs (hDPSCs) were examined as a cellular source for bone tissue engineering using an in vivo‐forming hydrogel, methoxy polyethylene glycol‐polycaprolactone block copolymer (PC),^[^ ^76^ ^]^ which forms in vivo hydrogels after subcutaneous injection into rats. Differentiated osteoblasts in in vivo‐forming hydrogel are identified by Alizarin red staining (ARS) and Von Kossa staining and are found to exhibit characteristic expression of genes like osteonectin, osteopontin, and osteocalcin (Figure 4D). The hDPSCs in injectable thermosensitive chitosan/b‐glycerophosphate/hydroxyapatite hydrogel also had good cellular compatibility and osteogenic differentiation in vitro.^[^ ^71^ ^]^ Another thermosensitive hydrogel, composed of poly(N‐isopropyl acrylamide), graphene oxide, and CS, serves as a scaffold for DPSCs, enhancing their osteogenic differentiation.^[^ ^77^ ^]^

Among those seed cells, the ASCs are a preferred cell source for clinical application as harvesting adipose tissue is easy and safe using local anesthesia.^[^ ^78^ ^]^ In order to enhance bone regeneration, a biocompatible thermogelling hydrogel, hyaluronic acid‐g‐chitosan‐g‐poly(N‐isopropylacrylamide) (HA‐CPN) with biphasic calcium phosphate and platelet‐rich plasma, was used as a 3D organic gel matrix for entrapping rabbit ASCs (rASCs).^[^ ^72^ ^]^ The hydrogel was a better injectable cell carrier for osteogenesis of rASCs with increased cell proliferation rate and alkaline phosphatase activity, enhanced calcium deposition (Figure 4E), mineralization of extracellular matrix, and upregulated expression of genetic markers of osteogenesis. New bone formation was observed when the hydrogel was implanted in critical‐sized rabbit calvarial bone defects.

The human TMSCs (hTMSCs) are another alternate source of adult stem cells for regenerative medicine.^[^ ^79^ ^]^ As with BMSCs, TMSCs contain a stromal population of cells that is self‐renewing and multipotent. TMSCs exhibit similar characteristics to BMSCs but are much more abundant, easier to harvest, and have a higher proliferation rate. In addition, TMSCs are less contaminated by erythrocytes than BMSCs.^[^ ^80^ ^]^ The MP solution loaded with hTMSCs formed a hydrogel at 37 °C within 10 s.^[^ ^36^ ^]^ Cells differentiated into osteoblasts within hydrogels in situ (Figure 4F).

The thermosensitive hydrogel can also be loaded with other cells for treating related diseases, such as human corneal endothelial cells derived from pluripotent stem cells^[^ ^81^ ^]^ and goat auricular chondrocytes.^[^ ^82^ ^]^ Various seed cells are being utilized in natural and synthetic materials, especially in thermosensitive hydrogels, and their osteogenic efficacy has been confirmed. However, the adhesion, proliferation, and differentiation of these cells are influenced by numerous factors of scaffold material properties, such as pore size, porosity, surface roughness, hydrophilicity/hydrophobicity, elastic modulus, and toughness. While further material improvements are necessary, a more pressing issue is the limited availability of seed cells due to human immune system constraints. These challenges must be addressed.

### 5.2 Carrying Bioactive Factors

**Figure 5:**
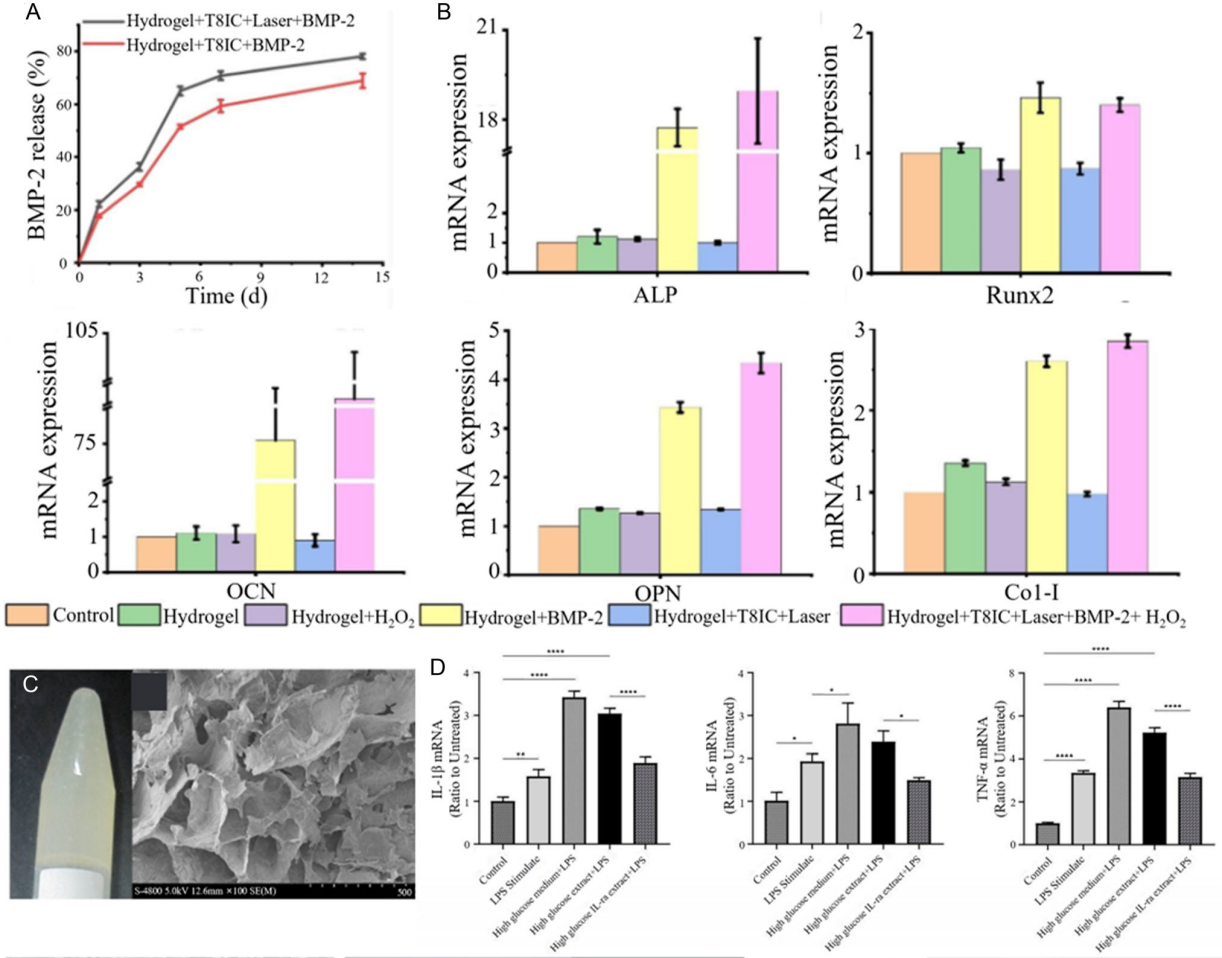
A) Cumulative release of BMP‐2. Reproduced with permission,^[^ ^84^ ^]^ Copyright 2023, BMC. B) mRNA expression of osteogenic markers (ALP, Runx2, OCN, OPN, and Col‐I). Reproduced with permission,^[^ ^84^ ^]^ Copyright 2023, BMC. C) Photographs and SEM photomicrographs (×100 magnification) of CS/BMP‐7/ORN hydrogel. Reproduced with permission,^[^ ^85^ ^]^ Copyright 2015, Faculdade de Odontologia de Bauru. D) Fold change of IL‐1β, IL‐6, and TNF‐α mRNA expression in the blank control, lipopolysaccharide (LPS) stimulated, high glucose medium + LPS stimulated, high glucose extract + LPS stimulated, and high glucose extract + LPS + IL‐1ra‐loaded CS/β‐GP/gelatin thermosensitive hydrogel groups (*n* = 3). Reproduced with permission,^[^ ^88^ ^]^ Copyright 2022, MDPI.

BMP exhibits remarkable osteo‐inductive ability by enhancing the recruitment and angiogenesis of osteoblast precursor cells. Injectable hydrogels with a 3D network structure have emerged as the preferred carrier for BMP in tissue engineering owing to their exceptional properties, such as high water absorption, injectability, biocompatibility, and biodegradability.^[^ ^83^ ^]^ An innovative injectable thermosensitive hydrogel was developed based on a 3D network, utilizing CS/β‐glutamine/gel as the carrier and incorporating BMP‐2 with T8IC nanoparticles and H_2_O_2_.^[^ ^84^ ^]^ This hydrogel undergoes a sol–gel transition at 45 °C within 2 min. Combined with mild photothermal therapy (45 °C), enhanced photodynamic therapy, and the sustained release of BMP‐2, the system demonstrated impressive bactericidal efficacy, osteogenic induction, and biosafety both in vitro and in vivo (**Figure** **5**A and B). There has also been a study that added rhBMP‐2 in alginate and pNIPAAm hydrogels.^[^ ^85^ ^]^ Meanwhile, BMP‐7 and ornidazole (ORN) were added into CS/β‐GP hydrogel. This hydrogel transforms into a nonflowable state at 37 °C and features a pore size of 171.5 ± 24.4 μM (Figure 5C). The released ornidazole effectively inhibits *Porphyromonas gingivalis*. When administered via syringe into the III‐degree root bifurcation lesion of beagles, the formulation significantly enhanced new bone formation at the site of the defect.^[^ ^86^ ^]^

Interleukin‐1β (IL‐1β) stands as a crucial multifunctional cytokine that has the capacity to encourage fibroblasts to secrete collagenase, interstitial lysase, and gelatin‐degrading enzymes. This, in turn, triggers matrix degradation, loss of connective tissue, and destruction of periodontal tissue. Additionally, it can stimulate osteoblasts to release plasmin and prostaglandin E2, promoting the differentiation of periodontal ligament mesenchymal stem cells into osteoclasts. This process ultimately leads to heightened inflammation and bone resorption. The biological activity of IL‐1β can be blocked by interleukin‐1 receptor antagonist (IL‐1ra), which binds to the IL‐1 receptor, subsequently reducing the expression of inflammatory factors like IL‐6 and TNF‐α, thus inhibiting the formation of osteoclasts.^[^ ^87^ ^]^ By carefully adjusting the ratio of CS, β‐GP, and gel, a thermosensitive hydrogel was developed.^[^ ^88^ ^]^ This hydrogel effectively loaded and stably released IL‐1ra for extended periods. When injected in situ into the alveolar bone resorption area, it demonstrated significant potential to alleviate hyperglycemia, suppress periodontal inflammation (Figure 5D) in diabetic periodontitis rats, and minimize alveolar bone resorption. Glycogen synthase kinase 3 beta (GSK3β) is a negative regulator in inflammation and bone homeostasis.^[^ ^89^ ^]^ A glycogen synthase GSK3 inhibitor (BIO) has been added to pyrophosphorylated Pluronic F127 and regular F127, effectively preserving alveolar bone and ligament and preventing periodontal inflammation.^[^ ^90^ ^]^

Currently, studies are still examining the incorporation of bioactive factors into thermosensitive hydrogels, such as the parathyroid hormone (PTH) or parathyroid hormone‐related protein (PTHrP) in poly(ethylene glycol)‐poly(ε‐caprolactone)‐poly(ethylene glycol) (PEG‐PCL‐PEG).^[^ ^91^ ^]^

Bioactive factors could be secreted by the body, and their significance in bone regeneration has been widely recognized. In situations where endogenous bioactive factors are inadequate to fulfill the immediate need for osteogenesis, exogenous bioactive factors, such as BMP‐2, BMP‐7, ornidazole, IL‐1 β, and GSK3 β, carried by various materials, become vital. However, exogenous growth factors face challenges like brief half‐life, instability, rapid enzyme degradation, and fast inactivation. These issues can be effectively addressed by modifying the scaffold material.

### 5.3 Others

The thermosensitive hydrogel scaffold not only carries seed cells and bioactive factors but can also transport additional substances such as sEV,^[^ ^92^ ^]^ exosomes,^[^ ^93^ ^]^ and matrix.^[^ ^94^ ^]^

The sEV serve as crucial paracrine mediators for MSCs in tissue regeneration, pivotal in intercellular communication. Their biological function is modifying the microenvironment under various pathological conditions by transferring biomolecules, including proteins, DNA, and miRNAs.^[^ ^92^ ^]^ The sEV derived from BMSCs can enhance periodontitis alveolar bone regeneration by boosting the migration and proliferation of periodontal ligament cells.^[^ ^95^ ^]^ The BMSC‐derived sEV and peroxide nanoparticles (CPO) were loaded into a thermosensitive adhesive hydrogel, which is composed of CS, poloxamer 407 (P407), and crosslinked HA (c‐HA). Additionally, ascorbic acid is incorporated to maintain redox and acid–base balances. Remarkably, this system can be injected using a 30‐gauge needle at 4 °C and rapidly transforms into a gel at 37 °C within 90 s. Both in vivo and in vitro experiments have demonstrated its excellent biocompatibility, effective inhibition of major periodontal anaerobic bacteria growth, reduction of periodontal pocket infections, and promotion of periodontal tissue regeneration.

M2 macrophage‐derived exosomes (M2‐Exos) can stimulate osteogenic differentiation of MSCs. In addition, the latest research suggests that M2 macrophage‐derived extracellular vesicles can be used as immune regulators to promote fracture healing. It was verified that the Pluronic F‐127(F127)/o‐nitrobenzyl alcohol‐modified hyaluronic acid (HA‐NB) hydrogel had a dense network structure, tissue adhesiveness, and dual sensitivity to temperature and light. It was liquid at low temperatures and can be solidified into a hydrogel at 37 °C. F127/HA‐NB loaded with M2‐Exos (M2Exos@F127/HA‐NB) exhibited good biocompatibility and achieved sustained release of exosomes for up to two weeks.^[^ ^93^ ^]^ The results of a rat cranial defect model showed that M2Exos@F127/HA‐NB had superior bone regeneration‐promoting effects.

Among the intelligent thermosensitive hydrogels, PEG‐PCL‐PEG (PECE) is a linear triblock polyester copolymer. Acellular bone matrix (ABM) with low immunogenicity has been successfully applied in multiple animal models, and the processed ABM informed the potential to support osteo‐induction. The injectable thermosensitive ABM/PECE composite presented promising potential in bone regeneration and benefited from incorporating the intrinsic osteo‐inductive ABM granules into PECE hydrogel.^[^ ^94^ ^]^ The transparent ABM/PECE solution became opaque and formed a nonflowing gel at the physiological temperature of 37 °C. The ABM/PECE composite was injected into the rabbit cranial defect and presented enhanced bone regeneration guidance.

## 6 Thermosensitive Hydrogel as a Delivery System in Maxillofacial Regeneration

The polymer network within the hydrogel, which features a high water content and physical or chemical crosslinking, exhibits exceptional capabilities in manipulating its physicochemical properties. These capabilities enable spatiotemporally precise control over the release of various drugs and therapeutics, while also providing effective protection for unstable drugs against degradation. Such attributes render it an ideal candidate for serving as an intelligent drug delivery system. Maxillofacial bone regeneration confronts various challenges, including anti‐infection measures and anti‐inflammatory efforts.^[^ ^96^ ^]^ A hydrogel component alone cannot meet these demands; consequently, a thermosensitive hydrogel serves as an outstanding delivery system and plays a pivotal role. Furthermore, the application of thermosensitive hydrogels in gene and drug delivery is emphasized.

### 6.1 Gene Delivery System

**Figure 6:**
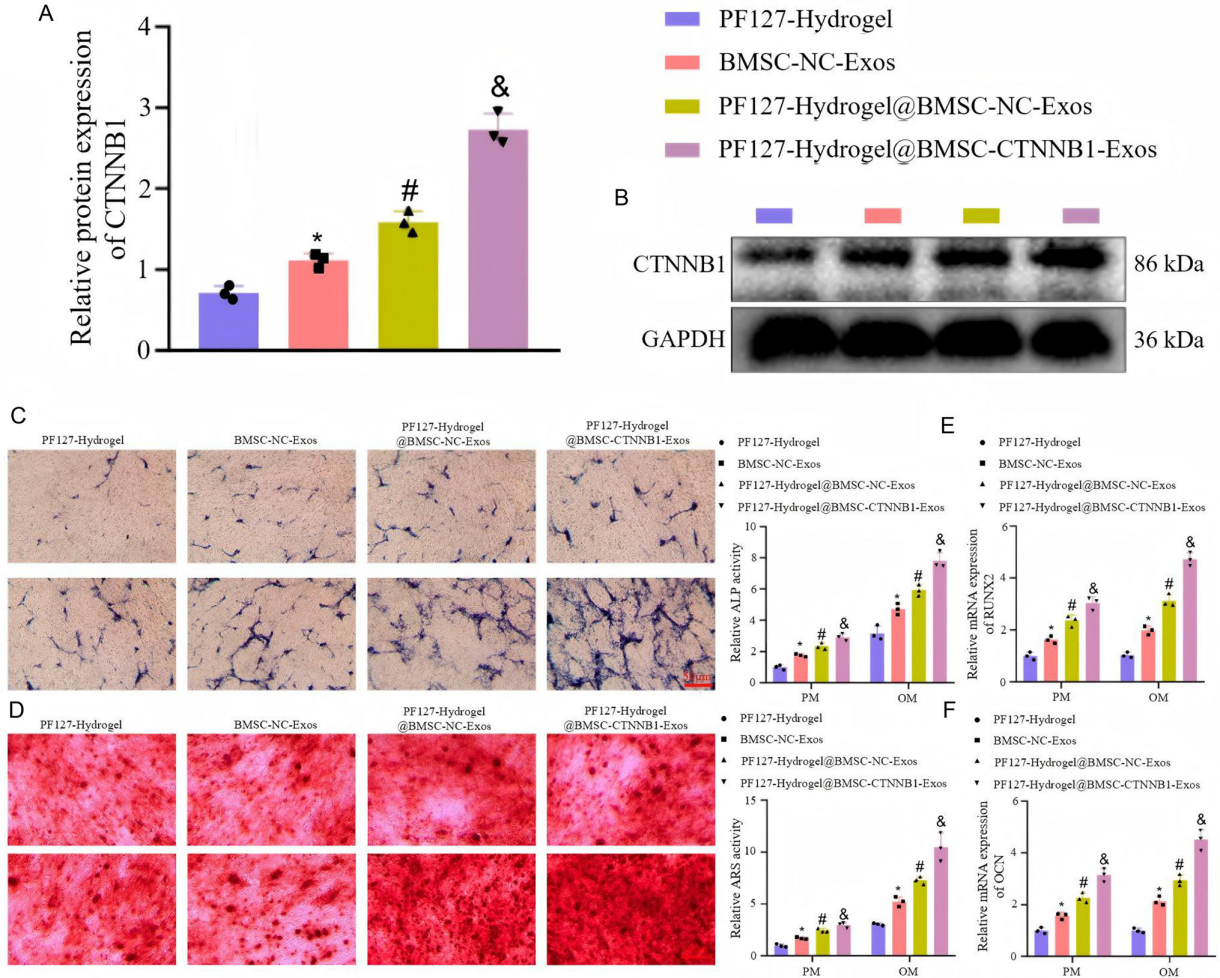
A,B) The protein expression of CTNNB1 in BMSC in response to PF127 hydrogel@BMSC‐CTNNB1‐Exos, as determined by western blot analysis. *P < 0.05 versus the PF127 hydrogel group. # *P* < 0.05 versus the BMSC‐NC‐Exos group. & *P* < 0.05 versus the PF127 hydrogel@BMSC‐NC‐Exos group. Reproduced with permission,^[^ ^97^ ^]^ Copyright 2023, MDPI. C) ALP staining and quantitative results of BMSCs cultured with proliferation medium (PM) and osteogenic medium (OM) in response to PF127 hydrogel@BMSC‐CTNNB1‐Exos. Reproduced with permission,^[^ ^97^ ^]^ Copyright 2023, MDPI. D) ARS and quantitative results of the BMSCs cultured with PM and OM in response to PF127 hydrogel@BMSC‐CTNNB1‐Exos. Reproduced with permission,^[^ ^69^ ^]^ Copyright 2023, MDPI. E) RT‐qPCR detection of RUNX2 expression in BMSCs cultured with PM and OM in response to PF127 hydrogel@BMSC‐CTNNB1‐Exos. Reproduced with permission,^[^ ^97^ ^]^ Copyright 2023, MDPI. F) OCN expression in BMSCs cultured with PM and OM in response to PF127 hydrogel@BMSC‐CTNNB1‐Exos determined by RT‐qPCR. *P < 0.05 versus the PF127 hydrogel group. # *P* < 0.05 versus the BMSC‐NC‐Exos group. & *P* < 0.05 versus the PF127 hydrogel@BMSC‐NC‐Exos. Cell experiments were independently repeated three times. Reproduced with permission,^[^ ^97^ ^]^ Copyright 2023, MDPI.

A thermosensitive Pluronic F127 (PF127) hydrogel was loaded with BMSC‐derived exosomes (Exos) (PF127 hydrogel@BMSC‐Exos).^[^ ^97^ ^]^ It was liquid at 4 °C, was in a semi‐solid colloid state at 37 °C, and could be gelated at 37 °C in about 43 s. CTNNB1 was predicted to be the key gene of BMSC‐Exos in the osteogenic differentiation of BMSCs, during which miR‐146a‐5p, IRAK1, and TRAF6 might be the downstream factors. The CTNNB1‐enriched PF127 hydrogel@BMSC‐Exos were constructed and implanted into in vivo rat models of alveolar bone defects. In vitro experiment data showed that PF127 hydrogel@BMSC‐Exos efficiently delivered CTNNB1 to BMSCs, which subsequently promoted the osteogenic differentiation of BMSCs, as evidenced by enhanced Alkaline Phosphatase (ALP) staining intensity and activity, extracellular matrix mineralization, and upregulated RUNX2 and OCN expression (**Figure** **6**).

Gene delivery can potentially introduce exogenous DNA into target cells, promising applications for treating numerous diseases. Despite this, the field still faces challenges in developing efficient and safe gene delivery vectors. Furthermore, the integration of these vectors with thermosensitive hydrogels remains sparsely documented. This area presents fertile ground for further exploration and advancement.

### 6.2 Drug Delivery System

A thermosensitive hydrogel was developed by adjusting the ratio of P407 and poloxamer P188 (P188), attaining an LCST of 32.62 °C and an optimal sol–gel time of 69.49 s.^[^ ^98^ ^]^ This hydrogel was loaded with egg yolk immunoglobulin (IgY) and antimicrobial peptide LL37, exhibiting a maximum drug release of 92.68% primarily through diffusion, good biocompatibility, and slow‐release capabilities. Using CS, β‐GP, and Gel to synthesize an injectable that underwent a sol–gel transition at 37 °C within 5 min and could load and continuously release aspirin and erythropoietin (EPO) to exert anti‐inflammatory and tissue regeneration effects lasting at least 21 days.^[^ ^11^ ^]^ In Wistar rats, the injection of this composite hydrogel into subperiosteal tissue demonstrated its ability to terminate inflammation and restore alveolar bone height.

Berberine could also be able to add in CS/sodium alginate (SA)/β‐GP hydrogels.^[^ ^99^ ^]^ These hydrogels gelled at 37 °C within 3 min, featuring pore sizes ranging from 10 to 130 μM. Their prolonged ability to carry and release berberine effectively inhibited inflammation, promoted the expression of osteogenic factors, and sustained anti‐inflammatory and osteogenic roles in periodontal tissue. Some scholars incorporated sodium bicarbonate into CS/Gel/β‐GP, observing that this addition accelerated gelation time and significantly enhanced the overall mechanical properties of the hydrogel.^[^ ^100^ ^]^ Furthermore, others have successfully integrated β‐tricalcium phosphate (β‐TCP) into CS/β‐GP, combining inorganic components with a chitosan hydrogel to effectively enhance the hydrogel's structure and performance.^[^ ^101^ ^]^

Focusing on the balance between Th17 and Treg cells by influencing macrophage polarization, an innovative therapeutic approach was introduced that involves dispersing folic acid‐modified quercetin microemulsions (FA‐Qu‐MEs) into a matrix hydrogel composed of P407 and pNIPAAm, creating a system known as FA‐Qu‐MEs@Gel.^[^ ^102^ ^]^ This system can be effortlessly extruded from a syringe and transition into a gel phase at 37 °C, allowing local injection into the periodontal pocket. This method enables continuous drug release, effective reactive oxygen species (ROS) clearance, regulation of the macrophage polarization cascade, restoration of Th17/Treg homeostasis, and, ultimately, the promotion of alveolar bone regeneration.

An MSN‐incorporated PDLLA (poly(DL‐lactide))‐PEG‐PDLLA (PPP) thermosensitive hydrogel with stepwise cargo release is designed to emulate the mesenchymal stem cell “recruitment‐osteogenesis” cascade for diabetic periodontal bone regeneration.^[^ ^103^ ^]^ The resulting nanocomplex solution could also perform sol–gel transition at 37 °C. During therapy, SDF‐1 quickly escapes from the hydrogel due to diffusion for early rat BMSCs recruitment. Simultaneously, slow degradation of the hydrogel starts to gradually expose the MSN for the sustained release of metformin, which can scavenge the overproduced ROS under high glucose conditions to reverse the inhibited osteogenesis of rat BMSCs by reactivating the AMPK/β‐catenin pathway, resulting in regulation of the diabetic microenvironment and facilitation of osteogenesis.

In addition, thermosensitive hydrogels can also be used in combination with scaffold materials. A study engineered a novel thermosensitive, sustained‐release hydrogel by amalgamating β‐cyclodextrin (β‐CD) with chlorhexidine (CHX) and decellularized extracellular matrix (dECM).^[^ ^104^ ^]^ They mixed this hydrogel with the nHA/PLA scaffold to promote bone regeneration. Drug delivery constitutes a crucial aspect of thermosensitive hydrogel applications due to its distinctive structure and gelation properties.

Due to the unique properties of the thermosensitive hydrogel, which exhibits a liquid state at low temperatures and transitions to a gel state at body temperature, drug‐loaded PLGA‐PEG‐PLGA thermosensitive hydrogel can be easily incorporated into the porous scaffold at 4 °C. Consequently, the composite scaffold material can be conveniently obtained at room temperature.^[^ ^105^, ^106^ ^]^

Thermosensitive hydrogel can also serve as bacterial delivery carrier in tumor treatment. At room temperature, it can effectively encapsulate engineered bacteria, which function as an artificial lymph island upon injection around the tumor.^[^ ^107^ ^]^ Hydrogel‐delivered engineered bacteria can also serve as a platform for monitoring tumors.^[^ ^108^ ^]^

## 7 Limitations of Clinical Application of Thermosensitive Hydrogel

A majority of the research on thermosensitive hydrogel in maxillofacial bone regeneration remains focused within the laboratory phase, with a noticeable absence of direct clinical application.^[^ ^109^ ^]^ The primary obstacles to its widespread use are issues related to long‐term biocompatibility, immune system response, scalability, and degradation rate. During the preclinical phase, researchers primarily investigated the biocompatibility of individual cells, including BMMSCs,^[^ ^95^ ^]^ RAW264.7 cells,^[^ ^99^ ^]^ and HUVECs,^[^ ^110^ ^]^ among others. Additionally, a limited number of studies performed hemolysis experiments to explore the biocompatibility of the hydrogel.^[^ ^103^ ^]^ During the animal experimentation phase, initial sampling observations were conducted without extended follow‐up.^[^ ^102^ ^]^ The study of bone immune regulation in bone regeneration remains predominantly confined to local investigations, with a notable absence of comprehensive systemic research.^[^ ^111^ ^]^

It usually takes 3–6 months or even longer to achieve stable osteogenic effects in the human body.^[^ ^112^, ^113^ ^]^ The compressive strength and Young's modulus of cancellous bone were 2–6 MPa and 0.1–0.3 GPa, respectively.^[^ ^9^ ^]^ The majority of thermosensitive hydrogels are primarily formed through physical crosslinking, such as chitosan and pNIPAAm, with limited strength. However, the performance of these hydrogels can be significantly improved by adjusting the gel network structure, incorporating dynamic bonds, and embedding nanoparticles.^[^ ^114^ ^]^

It is noteworthy that certain studies involve storing drugs for a specific duration, such as one month, and then comparing their release with newly prepared drugs.^[^ ^115^ ^]^ This experimental approach provides valuable reference insights for future experiments.

Preclinical studies are underway in craniomaxillofacial bone regeneration and repair, primarily utilizing rat and rabbit models. These investigations encompass a range of conditions, including periodontitis, calvarial defects, alveolar bone defects, mandibular bone defects, and bilateral class III furcation defects models. These models often incorporate comorbidities such as osteoporosis, diabetes, or local infections, allowing for a more comprehensive representation of the complexities involved in craniomaxillofacial bone repair (**Table** **2**). However, current animal models cannot adequately replicate the complexity of human maxillofacial environment, and further large animal studies, such as miniature swine and rhesus monkeys, need to be explored to further investigate the microbial exposure or functional loading of maxillofacial defects.

**Table 2:** Construction of various thermosensitive hydrogels and their application in maxillofacial bone regeneration.

Hydrogel composition	Modification	Load	Drug release	Transition temperature	Transition time	Pore size (porosity)	Mechanical properties	Degradation rate in vitro	Biocompatibility	Research model in vivo	Advantages for bone regeneration	References
CS/β‐GP/Gel	–	Aspirin/EPO	Aspirin(3 d, 86.6%) EPO(3 d, 69.4%) to 21 d)	37 °C	5 min	40–80 μm	Tensile strength 50.7 kPa at the strain of 76%	80% weight loss in PBS for 30 d	No cytotoxicity to BMSCs Good biocompatibility in nude mice and Wistar rats	Periodontitis model (Wistar rats, male)	Anti‐inflammation, bone regeneration	[11]
CS/silk fibroin	–	BMP‐2‐functionalized MgFe‐LDH/PDGF‐BB	BMP‐2(35 d(74.51%)), PDGF‐BB(7 d(80.03%)) Mg^2+^(35 d, 46.64 μg mL^−1^) Fe^3+^(35 d, 67.02 μg mL^−1^)	37 °C	146 s	10–100 μm	Compressive modulus ≈42 kPa	50% weight loss in PBS for 36 d	No cytotoxicity to hBMSCs and promote proliferation of HUVECs	Calvarial defect model (New Zealand White Rabbits)	Angiogenesis, bone regeneration	[17]
chitosan/β‐GP/gelatin	–	EPO‐EVs	EPO‐EVs (7 d)	37 °C	5 min	Loose and porous structure	Injectability	–	Higher cell viability of mBMSCs	Periodontitis with inflammatory bone loss model (C57BL/6 J mice, male)	Anti‐inflammation, bone regeneration	[134]
pNIPAAm	GRGDS	DMCs	–	37 °C contract 34 °C swell	<15 min	1618+108 μm (contract) 2398 + 211 μm (swell)	The cell volume decreased at ≈ 1.64 × 105 μm^3^ min^−1^ in our 3D time‐lapse study	–	No cytotoxicity to DMCs	Gel was implanted under the kidney capsule of an adult mouse	Mechanical compression induces cell differentiation	[35]
MPEG‐PCL	–	hTMSCs/osteogenic factors	–	37 °C	≤10 s	–	Viscosity 2.25 × 10^5^ cP	–	The viability of hTMSCs in MP was 85% after 5 d.	MP was subcutaneously injected into nude mice	Promote osteogenic differentiation of hTMSCs	[36]
CS/β‐GP	SFD	ZIF‐8@QCT	A gradual release pattern of quercetin was consistent with the hydrogel's degradation	37 °C	2 min	40 μm (55%–60%)	Adhesion strength 4.0 kPa	50% weight loss in artificial saliva for 14 d	No cytotoxicity to PDLSCs	Alveolar bone defect model with periodontitis (SD rats, male)	Antibacterial effect, immunomodulation, pro‐osteo‐/ angiogenesis, pro‐recruitment	[37]
PEG‐PLGA‐PNIPAM	MSN‐embedded core shell	MicroRNA222 /aspirin	MiR222/MSN(35 d, 80%) ASP(28 d, >80%)	33 °C	–	–	–	–	MiR222 can induce the differentiation of hBMSCs into neural‐like cells	mandibular bone defect model (SD rats, male)	Neurogenesis, bone regeneration	[61]
CS/GP	hydroxyapatite	hDPSCs	–	37 °C	15 min	10–20 μm	High injectability	–	Promote the proliferation of hPDSCs	–	Bone regeneration	[71]
HA‐CPN	BCP/PRP	rASC	–	–	–	–	–	–	Promote the proliferation of rASCs	Cranial bone defect model (rabbits, male)	Bone regeneration	[72]
Col/c‐PGA	–	mBMSC/BMP‐2	BMP‐2 (7 d, 88.9%)	37 °C	2 min	Open‐porous 3D interconnected structure	The viscosity at 25 °C is thrice the viscosity at 4 °C.	–	No cytotoxicity to mBMSCs	Calvarial defect model (nude mice, male)	Bone regeneration	[73]
CS‐PEO	–	hMSCs/rhBMP‐2	Rhodamine B (5 h, 80%) (14 h, 98%)	37 °C	Nearly instantaneously	–	Swelled to 2.0–2.5 times to an equilibrium in 13–19 h Compression strength 39.8 Pa	Completely degraded in 16–24 d in lysozymes solution	–	Calvarial bone defects (SD rats, male)	Bone regeneration	[74]
PLGA‐PEG‐PLGA	–	hPDLSC overexpressing PDGF‐BB	–	34.33 ± 0.57 °C	–	100 μm	Good fluidity	–	No cytotoxicity to hPDLSCs	Alveolar bone defect models (SD rats)	Bone regeneration	[75]
PC	–	hDPSCs	–	37 °C	10 s	–	Viscosity 2.31 × 10^5^ cP	–	No cytotoxicity to hPDLSCs	PC was injected into the subcutaneous dorsum of a SD rat	Bone regeneration	[76]
CS	pNIPAAm/graphene oxide	–	–	34.35 °C	–	Porous morphology	Reach the maximum swell level in 24 h Compression strength 0.4–9.7 MPa The storage and loss modulus for GH3 are 1.5 × 10^5^ Pa and 3.42 × 10^4^ Pa at 37 °C	13%–17% weight loss in PBS for 28 d	Good hemocompatible No cytotoxicity to hDPSCs	–	Enhancing hDPSCs osteogenic differentiation	[77]
Chitin	hydroxypropyl	–	–	17.5°C	<18 s (37 °C)	–	Injectability (1.2 mm needle) strength >300 Pa, swelling ratio 120% at 24 h	22.4% weight loss in PBS for 42 d 94.3% weight loss in lysozyme for 42 d	Low cytotoxicity to chondrocytes	Hydrogel encapsulated chondrocytes were administered by dorsal subcutaneous injection to nude mice	Cartilage regeneration scaffold	[82]
CS/β‐glutamine/Gel	–	T8IC/BMP‐2/ H_2_O_2_	BMP‐2(15 d, 78%)	45 °C	Within 2 min	Porous structure	The storage modulus and the loss modulus remained constant when the strain was below 100%	93% weight loss at 37 °C after 14 d	No cytotoxicity to MC3T3‐E1 cells without irradiation Cytotoxicity to MC3T3‐E1 cells with irradiation when the temperature was over 45 °C	Periodontitis model (C57BL/6 J mice, female)	Antibacterial effect, anti‐inflammation, bone regeneration	[84]
Alginate/pNIPAAm	–	rhBMP‐2	rhBMP‐2 (3–15 d)	37 °C	–	30–50 nm	Injectability (1.2 mm needle)	–	–	Alveolar bone defect model (New Zealand White rabbits, male)	Bone regeneration	[85]
CS/β‐GP	–	BMP‐7/ORN	BMP‐7 (14 d, 84%) ORN (80 min, 67%)	37 °C	–	171.5 ± 24.4 μm	Viscosities 50 Pa·s at 37 °C Swelling ratio 600% at 60 min	85% weight loss in PBS with lysozyme for 28 d	–	Bilateral class III furcation defects model (beagles, male)	Antibacterial effect, bone and cementum regeneration	[86]
CS/β‐GP/Gel	–	IL‐1ra	IL‐1ra (21 d, 83.23%)	37 °C	5 min	5–70 μm	–	75.20% weight loss in SBG for 56 d	No cytotoxicity to RAW264.7 cells	Diabetic periodontitis model (Wistar rats, male)	Anti‐inflammation, bone regeneration	[88]
Pluronic F127	Pyrophosphorylated	BIO	BIO (48 h, ≈99%)	37 °C	–	–	Shear‐thinning behavior	Completely degraded in 48 d in PBS solution	Slightly decreased cell viability of MC3T3‐E1 cells	Periodontitis model (SD rats, female)	Bone regeneration	[90]
PEG‐PCL‐PEG	–	PTH or PTHrP	PTH or PTHrP (14 d, 75%)	37 °C	–	Porous 3D structure with ordered spherical pores of homogeneous mesh size	–	–	Rare cytotoxicity to BMSCs	Orthodontic tooth movement model (SD rats, male)	Bone regeneration	[91]
F127/HA‐NB	HA‐NB	M2‐Exos	M2‐Exos (12 d)	37 °C	10 s	Porous morphology	Tissue adhesiveness Adhesive strength ≈8.5 kPa	–	Enhanced the proliferation and viability of BMSCs	Skull defect model (SD rats)	Bone regeneration	[93]
PEG‐PCL‐PEG	–	ABM	–	37 °C	–	–	Tube‐inverting method	–	No cytotoxicity to rat ROS 17/2.8 osteoblasts	Cranial defects model (New Zealand white rabbits)	Bone regeneration	[94]
CS/P407/c‐HA	–	sEVs/CPO/Ascorbic acid	O_2_ (7 d) sEVs (8 d)	37 °C	90 s	Irregular microcavities of porous structure	Injectability (30 gauge needle at 4 °C)	–	No cytotoxicity to BMMSCs	Periodontitis model (SD rats, female)	Anti‐inflammation, bone regeneration	[95]
Pluronic F127	–	CTNNB1‐enriched BMSC‐Exos	–	37 °C	43 s	–	–	–	–	Alveolar bone defects model (Wistar rats, male)	Bone regeneration	[97]
P407/P188	–	IgY/LL37	IgY (72 h) LL37 (72 h)	32.62 °C	69.49 s	Honeycomb‐like pore structure	–	–	–	Periodontitis model (SD rats, male)	Anti‐inflammation, bone regeneration	[98]
CS/SA/β‐GP		Berberine	BBR (21 d. 89.99%)	37 °C	3 min	10–130 μm	–	–	No cytotoxicity to RAW264.7 cells	–	Anti‐inflammation, bone regeneration	[99]
CS/Gel/β‐GP	NaHCO_3_	GM‐CSF/resveratrol	GM‐CSF (14 d) Resveratrol (14 d, ≈80%)	37 °C	5 min	Highly interconnected porous structure	Young's modulus 11.7± 0.2 kPa Swelling ratio ≈926%	80% weight loss in PBS for 14 d	No cytotoxicity to DCs	Subcutaneous injection (C57BL/6 mice)	Modulate DCs towards the tolerogenic phenotype and induce regulatory T‐cells under hyperglycemia	[100]
Chitosan	–	β‐TCP	–	37 °C	50 min	Porous structure	Linear visco‐elastic range (strain value 0.01%–1%)	20% weight loss in PBS and AS for 28 d (higher loss in lysozyme)	No cytotoxicity to MC3T3‐E1 cell line and HGF cells	–	Periodontal bone and soft tissue repair	[101]
P407/pNIPAAm	–	FA‐Qu‐MEs	Qu (24, 76.13 ± 1.61%)	32.6 °C	–	Pore structure	Excellent injectability	–	No cytotoxicity to RAW264.7 cells	Periodontitis model (SD rats, male)	Anti‐inflammation, bone regeneration	[102]
PDLLA‐PEG‐PDLLA	–	Met@MSN/SDF‐1	Met (30 d) SDF‐1 (14 d, 80%)	37 °C	–	–	Storage modulus was always larger than loss modulus under different conditions	Slow degradation for 30 d	No cytotoxicity to BMSCs No hemolysis	Mandibular periodontal bone defects and diabetes model (SD rats, male)	Diabetic periodontal bone regeneration	[103]
dECM	nHA/PLA scaffold	β‐CD/CHX	CHX (35 d, 66%)	37 °C	6 min	Porous structure (>60%)	Compressive strength 22.83 ± 0.17 MPa	–	No cytotoxicity to MC3T3‐E1 cells	–	Induce bone regeneration	[104]
GelMA/pNIPAM/pAAM	–	GO‐PL/berberine	Berberine (24 h, 60%)	45 °C	–	30.74–51.76 μm at RT, varied 14.5%–34.8% at 45 °C, porosity decreased 70.64%–84.35%	Young's modulus 10–21.7 kPa	–	17% decrease in cell activity of BMSCs by 5 d No cytotoxicity to HUVECs	Infectious cranial defect model (SD rats, female)	Antibacterial effect, promote angiogenesis, bone regeneration	[110]
Chitosan/gelatin/sodium bicarbonate/β‐GP	–	GM‐CSF/resveratrol	Storage did not affect the stability of resveratrol	37 °C	–	Porous structure	–	–	–	High‐fat diet and periodontitis model (SD rats)	Anti‐inflammation, periodontal healing	[115]
Carbopol 934 P NF/Pluronic F‐127	–	Lidocaine (0.25%–4%)/ metronidazole	Lidocaine (48 h, 68.7%–90%) Metronidazole (24 h, 20%)	Commenced at 23 °C Completed at 37 °C	–	–	Viscosity 2400 Pa·s	64%–81% gel dissolution in PBS for 35 d	Concentration‐dependent effect on cell viability of human gingival fibroblasts	–	Analgesic and antibacterial for alveolar osteitis,	[135]
Gelatin	Transglutaminase (TG) and tannic acid (TA)	His6‐T4L‐BMP2	His6‐T4L‐BMP2 (37 d, 4.5%)	37 °C	≈37s	3 μm	Compressive strength ≈105 kPa Storage modules 11 660 Pa great resilience, adhesivity, and flexibility	<20% weight loss in PBS for 28 d	No cytotoxicity to C2C12 cells	Calvarial bone defect model (SD rats, male)	Promote angiogenesis bone regeneration	[136]
methylcellulose	nHA	BMSCs	–	34.4 °C	60–75 s	50–200 μm	shear‐thinning elastic modulus 1600 Pa	15% weight loss in SBF for 5 d	No cytotoxicity to BMSCs	cranial bone defect model (SD rats, male)	Bone regeneration	[137]
MCPC/PLGA	Mn_*x*_Fe_3__‐*x*_O_4_ scaffold	VEGF/NGF	–	≈41 °C	–	Porous structure	–	60% weight loss in PBS for 21 d	Increased the viability of HUVECs, PC12 cells, and BMSCs	Osteoporotic calvarium defect model (SD rats, female)	Angiogenesis, neurogenesis, osteogenesis	[138]

“–” Indicates that it was not mentioned in the study.

Furthermore, the steep cost and intricacy of certain hydrogels pose additional challenges to their mass production and clinical utilization. Forthcoming research must tackle these impediments and enhance the outcomes in large animal models.

## 8 Conclusion

The maxillofacial bone exhibits a complex 3D curved structure, soft tissue coverage, powerful bite forces in specific areas, an abundance of local nerves and vessels, and a distinctive microbial environment.^[^ ^116^ ^]^ When selecting repair materials for bone regeneration in this region, it is imperative to prioritize high biocompatibility, precise fitting of bone defects, compatibility with the rate of bone remodeling in the maxillofacial area, and a certain level of antibacterial properties.^[^ ^117^ ^]^ Additionally, owing to the unique location, craniomaxillofacial bone repair demands not only bone volume compensation but also ensures that the newly regenerated bone tissue harmonizes morphologically and mechanically with the adjacent normal bone and soft tissue.^[^ ^118^ ^]^ This synchronization is crucial to preserve facial symmetry and esthetic contours.

Compared with other responsive gels, thermosensitive hydrogels consist of a 3D network structure capable of forming in vivo. By fine‐tuning the component proportions and modulating the interactions between the hydrogel's polymer chains and water molecules in the medium, a sol–gel transition can be induced at body temperature (37 °C).^[^ ^12^ ^]^ Due to their distinctive properties, these hydrogels have attracted increasing attention in the biomedical field. Furthermore, their fluidity in vivo, minimally invasive injectability, localized applicability, and controllable drug release make them highly advantageous for addressing irregular lacunar defects in the maxillofacial region. Despite numerous treatment strategies proposed by scholars for using thermosensitive hydrogels in maxillofacial bone defect repair and promising regeneration results observed in both in vitro and in vivo experiments, clinical application remains to be elucidated. Therefore, further research is imperative to develop a clinically viable thermosensitive hydrogel for widespread therapeutic use.

## Conflict of Interest

The authors declare no conflict of interest.
